# Aspects of Applied Chemistry Related to Future Goals of Safety and Efficiency in Materials Development for Nuclear Energy

**DOI:** 10.3390/molecules28020874

**Published:** 2023-01-15

**Authors:** Florentina Golgovici, Aurelia Elena Tudose, Diana Diniasi, Radu Nartita, Manuela Fulger, Ioana Demetrescu

**Affiliations:** 1 Department of General Chemistry, University Politechnica of Bucharest, Splaiul Independentei Street, No. 313, 060042 Bucharest, Romania; 2 Institute for Nuclear Research Pitesti, Campului Street, No. 1, P.O. Box 78, 115400 Mioveni, Romania; 3 Academy of Romanian Scientists, 3 Ilfov, 050094 Bucharest, Romania

**Keywords:** nuclear reactor, nuclear energy, generation IV reactor, zirconium alloy, austenitic steel, high entropy alloy

## Abstract

The present paper is a narrative review focused on a few important aspects and moments of trends surrounding materials and methods in sustainable nuclear energy, as an expression of applied chemistry support for more efficiency and safety. In such context, the paper is focused firstly on increasing alloy performance by modifying compositions, and elaborating and testing novel coatings on Zr alloys and stainless steel. For future generation reactor systems, the paper proposes high entropy alloys presenting their composition selection and irradiation damage. Nowadays, when great uncertainties and complex social, environmental, and political factors influence energy type selection, any challenge in this field is based on the concept of increased security and materials performance leading to more investigations into applied science.

## 1. Introduction

Understanding the basic chemical theories and principles to be applied in the fabrication and characterization of new materials with specific functions and properties represents a continuous challenge in materials development for many fields, such as energy [1], health [2,3], environment, and pollution [4,5]. Several aspects of applied chemistry are closely related to the development of new materials with controlled functions, particularly through a better understanding of materials properties, such as HEA.

Materials for energy depend on the energy type, meaning all traditional and renewable types [6,7], each of which is related not only to their properties but to a specific period of time and events.

For nuclear power reactors, materials history development is more complicated in our times due to the Chernobyl accident in 1986 and, more recently, the Fukushima power plant disaster that happened as a result of the magnitude 9.0 earthquake and tsunami that hit Japan on 11 March 2011 [8]. Many materials with large applications in nuclear technology such as Zr alloys, that have very low cross-section absorption of thermal neutrons, corrosion resistance and were indicated for the cladding of fuel rods, especially for water reactors, also found other uses, such as biomaterials, after the above mentioned events [9,10].

Despite previous disasters, as a low-carbon electricity source representing 25% of the total electricity generation in 2020 in Europe by fuel [11], both nuclear materials and nuclear power are treated nowadays with more interest in a conceptual frame of more safety and security in their service life [12,13]. Of course, it is a challenge for sustainable development [14,15], in the context of the simultaneous appearance of various technologies and methods supporting different specific domains [16], to be able to answer real-world problems in everyday life. It is a large scientific field with old and new methods of research design adapted for specific domains as a function of complex factors and their importance in a specific time with specific resources [17]. Energy has always been important for development, but its source, extension, and dynamic changes have been and will continue to be a function of complex social, economic, and political growth and decisions [18].

The studies were not able to find a direct correlation between gross domestic product (GDP) and energy production; however, considering GDP as an indicator of economic health, there are connections, even if it is not clear if energy drives gross domestic product increase or GDP growth drives the production of energy. It is important to notice that for the future generation of reactors [19,20,21], even though power efficiency was a main factor in selection, an electricity generation system choosing both traditional types and renewable sources in various proportions, safety use for a longer time and concept of clean energy has become more dominant. The Generation IV Reactor Integrated Materials Technology Program [19,22] includes several reactors with particular and common safety needs. The reactor systems are the Very High Temperature Reactor System (VHTR) [23], the Molten Salt Reactor (MSR) [24], the Gas-Cooled Fast Reactor System (GFR) [25], the Sodium-Cooled Fast Reactor System (SFR) [26], Lead-Cooled Fast Reactor System (LFR) [27], and the Supercritical Water-Cooled Reactor (SCWR) [28].

In the vision of this program, the new innovative systems that will work at high temperatures and aggressive environments (liquid Pb, molten salts) will require structural materials with high performance that can withstand these harsh conditions.

The present paper is devoted to several aspects of applied chemistry methods introducing strategies and recent advanced investigations of a new generation of nuclear materials that promote safety in the operation of the new nuclear reactor concepts. It is a narrative review, trying to objectively analyze some current trends in research on metallic alloys and highlight the best practices that promote safety and security. Nowadays, in a time of great uncertainties, revitalizing the concept of increased security for nuclear materials requires investigating fundamental and applied science for future generations of resources.

## 2. Relations between Microstructures and Alloys Performance of Future Generation of Nuclear Reactors

### 2.1. Zirconium Alloys in a New Concept of Safety

The development of nuclear reactor technology demands an improvement of the zirconium alloys through the optimization of the existing alloys or the development of new advanced alloys. Thus, a new generation of zirconium alloys (ZIRLO, OPT ZIRLO, and AXIOM in the US, M5 in France, E635 in Russia, HANA in South Korea, MDA in Japan, and N36 in China) [29,30,31,32,33,34,35,36] has been developed by alloying the current zirconium alloys with Sn, Nb, Fe, Cr, Ni, Cu, V, Si, etc., and the improvement of thermal treatments, showing better corrosion resistance in normal operation conditions [37].

**Figure 1:**
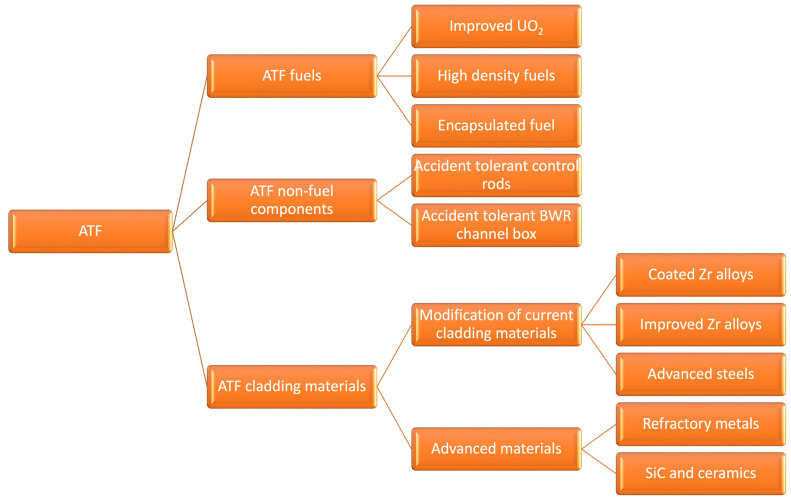
Introducing materials in the ATF concept.

The actual commercial zirconium alloys respond to the standards regarding corrosion resistance, creep, irradiation, and abrasion for normal operating conditions. However, water-side corrosion of zirconium cladding and hydrogen embrittlement are still key factors regarding the loss of cladding integrity [31,34,35,37,38,39,40]. Many studies have been conducted to obtain zirconium alloys with higher operational performance, therefore also increasing economic efficiency and operational safety [30,35,37,39]. After the Fukushima Daiichi accident, some research programs worked in developing new fuel systems with enhanced performance in normal and accident conditions, known as accident tolerant fuels (ATF) [37,41,42,43,44]. In this context, three research directions were developed: the optimization of chemical composition and manufacturing process of zirconium alloys, the development of new advanced alloys, and the development of coatings on the existing alloys [45]. The new fuels, cladding materials, and non-fuel components proposed as ATF are summarized in Figure 1.

At this moment, it is difficult to assess which research direction represents the best solution, but it is known that the replacement of zirconium alloys as cladding materials is a long-term solution, while the use of coated zirconium assures the improvement of the alloy performance without modifying the whole fuel concept of UO_2_/zirconium alloy [35,46,47]. Therefore, the coated zirconium alloys are the agreed technical solution for application in the nuclear industry in the near future.

Table 1 shows the leading institutes of each country and their ATF cladding options [29,48,49,50,51].

The development of coatings for zirconium alloys as ATF must be in accordance with technical specifications and specific performance requirements. The most applied coatings, which demonstrated [29,52,53,54] an enhanced performance of coated zirconium cladding, contain at least one of the following elements: Cr, Al. or Si. Table 2 summarizes the main candidate materials as coatings for nuclear fuel cladding [35,37,47,55,56,57,58,59,60,61].

The main results regarding the coatings development on zirconium substrate, and tested under normal and accident reactor conditions, are presented as follows.

The **FeCrAl coatings** are stable under normal pressurized water reactor (PWR) operation conditions, but at high temperatures (>900 °C), metal diffusion and consequently the formation of eutectics was observed [29]. Terrani et al. [62] developed, by hot isostatic pressing (HIP) method, a coating made from a FeCrAl layer and a 310SS layer. They observed the degradation of these two layers of coating at a temperature of 1300 °C. Other researchers [63] applied FeCrAl coatings with various compositions by magnetron sputtering on Zr-2 substrate, obtaining a substantial diminution of the corrosion rate at 700 °C. At 900 °C, however, the formation of Fe-Zr eutectic was observed. The same behavior of FeCrAl coating deposited on zirconium substrate by the cold spraying method, and tested at 1200 °C for 3000 s was observed by Park et al. [64]. It can be noted that the FeCrAl oxidation resistance is affected by the diffusion of metals and the eutectic reactions. A Mo layer between the FeCrAl coating and substrate can be applied to prevent the above problems [64,65]. Further experimental studies are necessary for the validation of FeCrAl coatings’ performance and reliability.

Experimental research has shown that the **CrN coatings** present good behavior under normal operating conditions and also under conditions specifically for high-temperature steam oxidation tests [66,67]. R.V. Nieuwenhove et al. [66] confirmed the excellent stability of a CrN coating deposited on a zirconium alloy substrate under irradiation conditions. Although, in accordance with Terrani et al. [47], recent results of LOCA tests on CrN zirconium alloy showed a very adherent coating, without any visible delamination, but the coating lost its integrity on the ballooning area of the cladding. Consequently, numerous cracks resulted on the cladding surface, limiting the capacity of the coating to provide protection to the cladding under high-temperature steam oxidation conditions [55].

**MAX phase carbides** (Cr_2_AlC, Ti_2_AlC, Ti_3_SiC_2_) have also been proposed as ATF coatings, but recent studies showed that their performances are not adequate. Roberts showed [68] that in the case of Ti-Al-C coating, various oxides and hydroxides are formed and for the Cr-Al-C coating the exfoliation of the substrate was seen during the corrosion tests. Ang et al. [69,70] tested a MAX material for irradiation in LWR conditions. The results indicated anisotropic swelling, visible cracks, and low mechanical properties for the Ti-Al-C series. Although the Ti-Si-C materials presented a better irradiation resistance than the Ti-Al-C series, a high reduction of Ti_3_SiC to TiC after irradiation tests was seen. Also, Tunes et al. [71] conducted tests on Ti-based MAX phases and they highlighted that these materials experienced phase decomposition and segregations caused by neutron irradiation at 10 dpa. Low dose irradiation of thin Cr_2_AlC films at room temperature caused the amorphization of the coating [72,73]. Although the thin Cr_2_AlC film showed good irradiation behavior at 450 °C [73], the higher hardness noticed after the irradiation test could increase the risk of cracking the coating and losing its protective character. Due to these points, the process of obtaining MAX phases is difficult. It is difficult to obtain pure MAX phases at low temperatures, and it is known that high temperatures induce changes in the zirconium microstructure [74,75,76]. Considering the results obtained up to this moment, it can be concluded that the MAX phase materials are not too suitable as coatings for ATF materials in LWRs [29].

**Metallic chromium** has a high melting point, good oxidation behavior at high temperatures, ductility at high temperatures, and a coefficient of thermal expansion similar to zirconium. These properties propose chromium as a coating material for zirconium alloys. Metallic chromium has a mechanical strength close to that of zirconium alloys, and a thin chromium film has a limited effect on the mechanical properties of cladding under normal operation conditions [55,77,78]. Due to the high hardness and strong adhesion of chromium to the zirconium substrate, the chromium-coated cladding presented substantially improved wear resistance compared to uncoated zirconium alloy [55,79]. Therefore, the degradation probability of cladding due to grid-to-rod vibration and debris friction during reactor operation is limited. It was reported that chromium coatings enhanced the corrosion resistance of zirconium claddings tested in an LWR environment [55,79,80,81,82], which is advantageous regarding reactor life extension and the burnup increase of a fuel nuclear element. Also, it was observed that the chromium-coated zirconium alloy in a primary cooling water environment has an increased chemical resistance, thus improving the flexibility of operations under actual water chemistry limitations [79]. The results of ion and neutron irradiation highlighted that Cr coatings with body-centered cubic structures (BCC) showed certain irradiation stability [83,84]. The coatings prepared by cold spraying methods present particular resistance to irradiation degradation [85]. An overall assessment of Cr coatings in ex situ conditions confirmed a good adhesion to the substrate, which allows for maintaining the coating integrity in normal operating conditions. Regarding the resistance of Cr coatings under steam oxidation and high temperature conditions, it was seen as an excellent performance. The oxidation rate for a coated zirconium sample was lower by at least one order of magnitude than an uncoated sample [86,87]. More than that, it was observed that Cr coating reduces the cladding hydrogen absorption and considerably minimizes the risk of hydrogen-induced embrittlement. As a result, the cladding maintains its mechanical properties for a longer period and doubles the oxidation time before the cladding degradation during quenching [82,86,87]. The use of coatings is supposed to raise the peak cladding temperature from 1200 °C, as is indicated in the present standard, to 1300 °C [47]. Furthermore, chromium depositions were found to reduce the high-temperature creep rate of Zirconium cladding and increase the bursting time in the early LOCAs by two to three times [88,89]. These modifications can eliminate the problem of a blocked coolant channel in a nuclear fuel sub-assembly. The protection provided by chromium coating protection was observed keeping its adhesion property, even in the vicinity of severely deformed bursting areas. Generally, a chromium coating remarkably enhances the stability and integrity of Zr alloys claddings during severe accidents, providing more “copying time” for an operator.

It should be noted that besides the great attention paid to the development of chromium coatings, the KAERI Institute of South Korea proposed the concept of ATF cladding with a modified surface by incorporating Cr or CrAl coatings and an oxide dispersion-strengthened (ODS) surface treatment [78,90]. The objective was the simultaneous improvement of steam oxidation at high-temperature resistance and of mechanical properties at high temperatures of zirconium cladding. Assessment of the CrAl performance based on the tests in out-of-reactor conditions showed excellent behavior both in normal and accident conditions. The zirconium alloy subjected to ODS treatment at 380 °C presented an enhanced anti-ballooning and bursting performance in a LOCA simulation test compared to the performance of the coating without ODS treatment [91]. Additionally, results from tests performed in the Halden Research Reactor showed that these ATF materials with applied ODS surface treatments present stability in operation [92].

### 2.2. Austenitic Steel for Better Performance in Generation IV Reactors

Nowadays, increased energy demand and the necessity of controlling worldwide CO_2_ emissions have made the option of energy production from nuclear reactions by fission and/or fusion very attractive [31,93,94,95,96,97,98,99,100]. As a consequence of the development in the field of energy production by fission reactions, Generation IV nuclear reactors became a real international challenge as a safe, innovative, durable, and economical method for energy production. The goal of advanced nuclear systems is to improve the performance of current reactors and fuel cycles, in terms of better economic efficiency, enhanced safety, waste minimization, and resistance to proliferation [101,102]. The Generation IV International Forum (GIF) selected the six innovative nuclear fission reactor concepts based on various aspects that address safety and environmental issues: the Sodium-Cooled Fast Reactor (SFR), the Lead-Cooled Fast Reactor (LFR), the Gas-Cooled Fast Reactor (GFR), the Very-High-Temperature Reactor (VHTR), the Molten Salt Reactor (MSR), and the Supercritical Water-Cooled Reactor (SCWR) [102]. These advanced nuclear reactor systems operate at much higher temperatures and utilize different coolants, including liquid metals such as sodium and lead-bismuth, high-pressure helium gas, molten salts, and water in the supercritical state. The reliability of nuclear power systems depends on the performance of structural materials, the degradation of which may be caused by several aspects of the harmful environment within a nuclear reactor. Structural materials used in the cores of advanced reactors will be subjected to a combination of high temperature, high-dose neutron irradiation, and stress. Under these conditions, the mechanical properties and irradiation resistance of conventional reactor structural materials can no longer meet the service requirements [103,104,105]. Degradation of materials in these harsh environments can lead to reduced performance and, in some cases, even failure [31,106,107,108].

SCWR is one of the six advanced concepts selected by GIF; it uses water above its critical thermodynamic point (374 °C, 22.1 MPa). The SCWR system is a logical evolution of the LWR (Light Water Reactor) [95]. Building SCWRs is based on two proven technologies: light water-cooled reactors (LWRs), which are the most common power-generating reactors in the world, and fossil fuel supercritical plant systems. Compared to LWRs, SCW reactors have the following advantages: the use of a single high enthalpy phase coolant removes the boiling crisis, discontinuous regimes of heat transfer in the core, and it increases performance safety [93,109,110], and the elimination of some very expensive components, such as steam generators and dryers, leading to nuclear power plant (NPP) simplification. Due to the high thermal efficiency (up to 50%) compared to LWR systems (about 33%) and due to the simplification of the plant, the SCWR is an advanced nuclear system [111]. Thus, SCW reactors meet the economic safety and sustainability criteria considered by GIF. Although these reactors are more efficient, water at high operating temperatures and pressures is a much more aggressive environment to both in-core and out-core components of the reactor compared to the coolant used in conventional water-cooled reactors [93,110,112]. Because the environment existing in a water-cooled supercritical reactor is unique, currently there is limited data on the behavior of materials under these specific conditions [101]. In this sense, one of the major challenges of developing such a reactor is the selection of suitable materials for the use of in-core and out-core components of the reactor. When discussing the selection of materials for SCW reactors, the thermophysical phenomenology of supercritical water must be considered [113]. Candidate materials for the construction of the internal components of the SCWR must exhibit resistance to corrosion and radiation, and have very good mechanical properties, and dimensional and microstructural stability.

The corrosion performance of candidate materials for the construction of SCW rectors can be affected by the material composition and structure, SCW temperature and pressure, water chemistry, and exposure time [114]. Although there are some studies on the effects induced by such extreme conditions on different alloys, a final decision has not yet been made regarding the structural materials used for the construction of SCW reactors.

Development, testing, and selection of suitable materials for both nuclear fuel cladding and internal components are of great importance in designing SCW reactors. The main requirement of such materials is to be able to keep their integrity not only during normal reactor operation but also during abnormal transient events. There are mainly three directions in the field of research and development of structural materials for SCW reactors:

- The selection of the best performing materials from among those already available;
- The development of new materials with better characteristics than the existing ones (ODS—steels hardened by oxide dispersion);
- The modification of the existing materials in order to improve their characteristics or performance through various surface coating methods.

Depending on the corrosion resistance in conventional nuclear reactors, boiling water reactor (BWR), pressurized water reactor (PWR), and Canada Deuterium Uranium (CANDU), there are three classes of candidate alloys for the construction of SCW reactors: ferrite-martensitic steels (HT-9, T91, T92 and HCM12A), austenitic stainless steels (304, 304L, 316, 316L, 310), and Ni-based alloys (IN718, IN625).

The austenitic stainless steels are the chromium-nickel 300 series and chromium-nickel-manganese 200-series steels. These types of steels are austenitic, nonmagnetic, do not harden by heat treatment, and have the best high-temperature strength. Austenitic stainless steels (SS) are widely used within the cores of LWRs. The 304 and 316 stainless steels were used in the PWR and BWR cores. The corrosion resistance of austenitic stainless steels is better than that of both ferritic and martensitic stainless steels [115].

Austenitic stainless steels were extensively used for core components at the beginning of the light-water reactors technology in the 1960s [116]. They have also been considered as structural components in innovative nuclear fusion test reactors due to the very good results they have obtained in applications in extreme environments [117,118,119]. The 300 series steels (Fe-Cr-Ni alloy) have good corrosion and mechanical properties for applications at high temperatures, making them viable to be applied in aeroengine parts, turbochargers, oil and gas pipelines [115,116,117,118,119,120,121,122,123,124,125,126,127,128,129,130,131], and as structural components in nuclear reactors [116,117,132,133]. The 304 SS is one of the austenitic steels that is most used in nuclear reactors. This material is known as 18/8 because it has a composition of approximately 18 wt% chromium and 8–10 wt% nickel [115].

**Figure 2:**
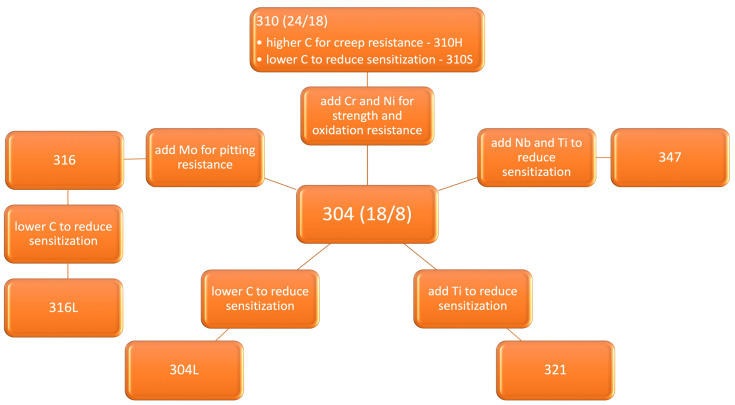
Compositional changes of 18/8 austenitic stainless steel to improve performance [134,135].

In Figure 2 are presented some compositional modifications of 18/8 austenitic stainless steel to obtain special properties [134,135].

The severe operating conditions of advanced nuclear systems have led to the continuous improvement of the performance of standard commercial stainless steel grades to operate at high temperatures and radiation by changes in the composition of the alloys. This was achieved either by the addition of interstitial elements such as C and N or by substitution such as Mn, Co, Ti, Nb, V, W, Cu, and Al. Thus, a new series of steels have been developed such as 304L, 304LN, 316L, 316LN SS, and 310. Regarding core applications in intense radiation environments, stainless steels such as 20% cold worked 316 SS, D9, and D9I have been developed to yield high burnup and to triple the lifetime of the core components of fast reactors [136].

The effect of alloying or impurity elements, such as carbon, silicon, manganese, and phosphorus, on properties such as swelling has been studied by Garner [137]. In these stainless steels, silicon and manganese are present to aid in processing. Silicon is added to suppress void swelling [138] without affecting other properties of the stainless steel. The steels that have added Mo (316) are stabilized with Ti (321) or Nb (347), having reasonably good elevated temperature strength and creep resistance [139]. The Mo addition in 316 SS is the main compositional difference between 316 SS and 304 SS; thus, improved corrosion properties are obtained. In the case of the Canadian Deuterium Uranium Pressurized Heavy Water Reactor (CANDU–PHWR), the calandria vessel, condenser piping, and preheater piping are, however, made from 304 SS.

The austenitic 304L and 316L grades were selected as the most effective in corrosive environments [21,140]. Until now, the corrosion tests were performed under simulated conditions of primary PWR and BWR at temperatures up to 320 °C [140] and in simulated conditions of CANDU NPP at temperatures up to 310 °C.

Due to their high resistance against creep and corrosion, as well as their good performance against radiation, austenitic stainless steels have attracted particular interest for use in applications at higher temperatures and aggressive conditions (such as 550 °C and 250 atm). Among these, the 310H-type is a 300-series chromium-nickel austenitic stainless steel that starts with 304-type stainless steel, with chromium and nickel additions for strength and oxidation resistance. This austenitic steel with a crystalline structure of the FCC type [141] combines excellent properties at high temperatures with good ductility and weldability, being designated to be used in environments with temperatures between 800 °C and 900 °C. Also, the austenitic 316L SS is considered to be another candidate material for the construction of internal components in SCWR, even if the corrosion tests performed in water at supercritical temperatures reveal a degradation tendency of the oxide film formed by the spallation process.

Applying protective coatings is considered a possible solution to increase the performance of materials. Currently, for high-temperature applications, there are several deposition techniques of thin layers, but the most used are physical vapor deposition (PVD), chemical vapor deposition (CVD), thermal spray coating, pack cementation, pulsed laser deposition, electrodeposition, plasma electrolytic oxidation (PEO), sol-gel, cold spray, and hot dipping. Vapor-deposited coatings are dense and the applied layer is thin. These types of coatings lead to a reduction in the amount of moisture or gas that can penetrate the material. Therefore, these types of coatings would be ideal to reduce the corrosion of materials in a nuclear environment. This applies to current LWR reactors (BWR, PWR, and CANDU) but also to Generation IV reactors such as SCWR or metal-cooled reactors liquids [142,143,144].

Several materials have been deposited on the surface of stainless steels used in the nuclear industry over time, using various deposition techniques: CrN, CrAlN, TiN, TiAlN, Al, Al_2_O_3_, FeCr, NiCrAlY, Ni_20_Cr, Ni_50_Cr, Ni_20_Cr_5_Al, Ni_5_Al, FeAl, FeAl-Cr(MoSi), Fe(-13, 15, and -20%)Al, (Ta_2_O_5_)_0.04_, (CeO_2_)_0.96_, (MgO)_0.01_, ZrO_2_, (ZrO_2_)_0.99_, and O [93,94,112,145,146,147,148,149,150,151].

Following the analysis of the information from the scientific literature, it was found that CrN and NiCrAlY coatings deposited through various PVD and thermal spraying techniques have a high resistance to oxidation at high temperatures, making them thus promising coating materials for deposition on the stainless steel surface. High oxidation resistance is due to the formation of a corrosion-resistant layer (Cr_2_O_3_, Cr_2_O_3_ and Al_2_O_3_) on the material surface that protects both the basis material (substrate) and the deposited layer.

There are a few studies regarding the behavior of the CrN_x_ and NiCrAlY coatings and the oxides formed on their surfaces after exposure to high temperatures and pressures. Tudose et al. [152,153,154] investigated the corrosion behavior of uncoated 310H SS and CrN_x_-coated 310H SS in water at supercritical temperatures (550 °C and 25 MPa) for up to 2160 h. Also, the performances of uncoated and NiCrAlY-coated 310H SS in water at a supercritical temperature of 500 °C have been studied by Huang et al. [146]. It was observed that the NiCrAlY coating has excellent oxidation resistance, corrosion resistance, and wear resistance. More information about the properties of chromium nitride is also presented in detail in a previous paper [153].

According to the literature data [155,156,157] published until now, CrN is considered to be the best coating for high temperature and high pressure operation due to its excellent mechanical properties such as high hardness, high ductility, low friction coefficient, higher toughness [145,158,159], wear resistance [145,160,161], and oxidation resistance (this type of coating begins to oxidize at 700 °C) [162,163].

Among the major requirements and challenges for the materials proposed for the new generation of nuclear reactors, the following can be mentioned [37]:

- The capability to coat or treat full-length cladding tube/table with the desired microstructure and an acceptable cost;
- Relatively low fabrication temperature to avoid changing the microstructure of the underlying based alloy;
- No or little negative effect on neutron economics;
- Good thermal properties;
- Good corrosion and irradiation resistance under normal operating conditions;
- Good mechanical properties;
- Improved resistance to high-temperature steam or air under accident conditions.

When selecting the coating, two conditions should be taken into account: the application technique (not to modify the general properties of the substrate) and the operating environment [142]. Regarding the operating environment, the coatings must withstand the working temperature and be compatible with the aqueous or non-aqueous environment (the coating must not dissolve or oxidize over time).

The use of austenitic steels as the principal class for in-core structural materials was taken into account for all GEN IV reactors regardless of the type of coolant.

For example, in the case of LFR, the austenitic steel AISI 316L(N) is considered a candidate for the reactor vessels, inner vessel structures, primary pumps, and steam generator while 15–15 Ti (named D9 or DIN 1.4970) was chosen for the cladding and other fuel element parts [164].

Molten lead is a promising coolant for one of the new generations of nuclear reactors (LFR) due to its high atomic mass, low neutron absorption cross-section, high boiling point, low vapor pressure, and good heat transfer properties. However, liquid lead is corrosive for structural materials and fuel cladding [165].

One of the key limitations of the design and application of liquid metal as a coolant in advanced nuclear reactors is the ability of structural materials to resist corrosion. Corrosion in liquid metals has been recognized as one of the most serious problems in the use of this coolant at high temperatures and has been studied widely in the last few years.

Except for oxidation and dissolution [166], the most important degradations in liquid lead are caused by: flow accelerated corrosion (FAC) [167], grid-to-rod fretting [168], and HLM-assisted loss of mechanical properties, such as liquid metal embrittlement (LME) and low cycle fatigue [169], or liquid metal assisted creep [170].

Generally, austenitic stainless steels are claimed as excellent corrosion resistant materials being less sensitive to oxidation than martensitic steel.

Thus, in saturated oxygen liquid metals, in the temperature range of 450–500 °C, the AISI 316 steel shows better resistance to oxidation than T91, which is attributed to the higher content of chromium in the austenitic steel in comparison with the martensitic steel [171]. The formed oxide layer is a duplex scale type. This duplex oxide layer is composed of a Fe-Cr spinel oxide layer that is in contact with the steel. Above this layer, a porous magnetite layer is observed which is in contact with the liquid alloy. Both layers have approximately similar thicknesses and the interface between them corresponds to the original interface steel/Pb [12]. Nevertheless, under stress, the external magnetite layer can crack easily [12].

Instead, in liquid lead with low oxygen content, austenitic stainless steels present a selective dissolution of nickel, which may result in the local transformation of austenite into ferrite in the depleted surface zones. Oxygen is required in the coolant at a sufficient level to allow for the formation of an oxide layer on the surface of structural steels (passivation), which minimizes the dissolution of the alloy elements (Fe, Cr, Ni, and Mn) in the coolant. On the other hand, oxygen saturation in the coolant must be avoided in all the parts of the reactor system to prevent the deposition of PbO, which may have plugging effects on the circulation, especially in the cold points (i.e., the heat exchanger and steam generator). From these causes, the oxygen concentration in liquid lead should be in the range of 10^−6^–10^−8^ wt.% [172].

After many years of research, it was established that the solution to prevent interaction between liquid lead and metal consists of finding protective methods for the structural materials. Some of the most suitable solutions have been based on the use of inhibitors [172] or corrosion resistant bulk alloys (FeCrAl steels) [173].

Another new method is to set a barrier between the steel matrix and the environment. The principal requirements of these barriers are to be adherent to the steel substrate and mechanically performant.

In this sense, ceramic coatings, like aluminum oxide, are a promising option because alumina is essentially insoluble in lead, and because it is compatible with a wide range of thermodynamic conditions in terms of temperature and oxygen content.

In time, several materials were deposited on stainless steel surfaces using different techniques: SiC, Si_3_N_4_, AlTiFe, FeAl (by CVD, PVD), FeCrAlY (by TS), TiN (by CVD), CrN (PVD), Al (pack cementation), Al_2_O_3_ (by pulsed laser deposition-PLD, atomic layer deposition-ALD, flame spraying), Ta (electro-chemical, vapor phase), Mo (plasma spraying, flame spraying, chemical deposition), Nb (magnetron sputtering, electron sputtering, galvanic deposition), WC (plasma spraying), Ti (plasma spraying), and AlTiN (high power impulsive magnetron sputtering) [173,174].

Among the materials that showed positive behavior, the most used are the aluminum-containing coatings. Numerous corrosion tests of aluminum oxide coatings have demonstrated that they are able to prevent both dissolution and rapid oxidation of steels in molten lead.

These coatings develop protective and stable alumina layers based on the Al selective oxidation mechanism, when exposed to liquid lead containing small amounts of oxygen.

Some studies regarding ceramic coatings using detonation gun thermal spray presented Al_2_O_3_ deposited in a wide range of thicknesses (from a few up to hundreds of micrometers), providing coatings with extremely good adhesive strength, low porosity, and some compressive residual stresses. Various austenitic steels coated with alumina using the PLD technique were also tested in liquid lead. PLD uses high power laser pulses to enable a laser ablation process that converts the coating precursors into plasma. When this plasma is directed toward the substrate surface, a thin and compact film is deposited. PLD is a quite versatile technology that allows for depositing thin films of a wide range of materials. By adjusting the deposition parameters, the coating microstructures can be manipulated ranging from a dense and compact film to a columnar and porous structure [175].

However, the efficiency of these coatings is limited by their lack of self-healing ability in the case of damage, which is very likely because of the difference in lattice parameters and thermal expansion coefficients of aluminum oxide and steel, as well as the brittleness of the oxide.

To obtain the desired characteristics of the coatings, these techniques can be modified or, in some cases, combined.

It was established that, in the case of coatings, they are useful because the deposition of a sublayer of Fe-Cr-Al on the steel under the aluminum oxide slows down the diffusion of steel components into the coating and reduces the risk of the coating cracking by decreasing the stress level in it [175].

In addition, when interacting with liquid lead at high temperatures, this substrate in turn develops an oxide film enriched with aluminum oxide, which is very effective in preventing steel corrosion.

Therefore, even if the aluminum oxide coating can exfoliate during contact with liquid lead, the metal matrix continues to be protected. Thus, the use of an aluminum oxide coating with sublayers that contain Al in their composition promises an increase in the lifetime of fuel elements for reactors with a heavy liquid metal coolant [176,177].

Another way to protect the surface of steels is to add aluminum to the alloy composition, to develop a protective alumina layer that self-heals through contact with the oxygen from liquid lead. These are so-called alumina-forming austenitic (AFA) steels, which can be considered materials even for other types of advanced reactors (i.e., SCWR) [178]. The selection of aluminum to form the oxide (Al_2_O_3_) imposes precise tuning of the chemical composition of the steel. It needs to add a minimum content of Al to form the alumina layer and, at the same time, to maintain the austenitic phase of the matrix, since aluminum is a ferrite promoter.

AFA steel was developed on the basis of High-Temperature Ultrafine Precipitate Strengthening Steel (HTUPS), which combines the advantages of high-temperature ultrafine precipitation, reinforcing, and forming the Al_2_O_3_ protective scale at a high temperature. The first AFA alloy is HTUPS 4 with Fe-14Cr-20Ni-2.4Al wt.%, which forms a continuous and external Al_2_O_3_ protective scale at 800 °C in the air and has good creep resistance. On this basis, many AFA alloys were developed by adding different contents of alloying elements according to the target. According to the main function of the alloying elements, it can be divided into basic elements, antioxidant elements and precipitates elements [179].

The basic elements are iron, nickel, chromium, manganese, and silicon. Iron is the basic element of the iron base alloy, nickel is the basic element to ensure the austenite structure, chromium is a common element in stainless steel and can promote the formation of alumina and manganese, and silicon can improve the fluidity of the alloy. The main corrosion-resistant element is aluminum. In addition to aluminum, elements of stabilizing alumina scale are chromium, niobium, carbon, and boron, and the active elements hafnium and yttrium improve the adhesion of the oxide scale. Elements with precipitation mainly refer to the elements that can help with the precipitate phase when the alloy is in service. In AFA alloy, niobium, titanium, vanadium, thallium, tungsten, molybdenum, copper, carbon, and boron can form metal carbides (MC) [179].

Surface coating technology can be a possible solution, but particular attention needs to be paid to certain aspects: self-healing, the bonding force between the coating and the substrate should be strong enough to ensure that the coating will not easily fall off; the formation temperature of the coating should not be too high to avoid the structure coarsening and performance deterioration of the steel substrate.

The main issue with ceramic coatings in general is their lack of self-healing properties. The aggressiveness of the lead coolant is combined with high temperatures and intense neutron radiation fields, which ultimately yield ever-growing stresses and strains, as found for fuel cladding under LFR conditions. The greatest challenge under such conditions is to guarantee that the structural integrity of ceramic coatings is never compromised, meaning that a coating must be able both to withstand the expected radiation damage exposures, and to accommodate the stresses and strains imposed by the fuel cladding without cracking or delaminating. This is an important requirement to guarantee corrosion resistance.

From the literature data, it was concluded that even a thin coating (a few micrometers) can protect and limit corrosion without causing LME issues [179,180].

Furthermore, sometimes the presence of the coating could improve some mechanical properties in liquid metal because of a reduction of contact between the steel and the liquid metal.

Additionally, the construction cost of nuclear power plants and implicitly the cost of the materials used is another important aspect. However, the estimates are uncertain, ranging between $5500/kW and $8100/kW [181]. A report from 2020 shows that for all the evaluated Gen III/III + I projects, the ex-post construction cost was higher than the initial announced budget. While the initial budged was in the range of $1828/kW–$4300/kW, the ex-post construction cost was in the range of $2410/kW–$8620/kW. From these costs, approximately 60% of the total cost is given by materials and components, the reactor systems, and the turbine plant equipment [182].

## 3. High Entropy Alloys—Materials for Future Nuclear Reactors

For a long time, alloying involved using a primary element with desirable properties as the matrix and other elements, typically in lower concentration, to add new properties or enhance the existing ones [183].

This method produced a limited number of potential alloys, most of which were studied. The first articles that examined alloys with multiple components appeared in 2004, as a result of researchers turning their attention to the vast undiscovered region of multicomponent phase diagrams, which consisted of elements combined in near-equiatomic or even equiatomic combinations [184].

In an article published by Cantor et al., the researchers looked at alloys made up of 16 and 20 elements in equal proportions, noting that some of those elements formed a single phase solid solution [185]. Separately, Yeh et al. put up a justification for the creation of these multicomponent alloys in the same year. They postulated that because mixing reduces the probability of intermetallic compound formation, random solid solutions will typically develop in most situations. This theory led to the designation of these alloys as high entropy alloys (HEA) [186].

The following equation, which takes into account the entropy of mixing (Δ*S_mix_*) for a completely random mixing, can be used to classify an alloy as high, medium, or low entropy:

(1) $$\Delta S_{mix}=-R\sum_{i=1}^{n}\left(c_{i}lnc_{i}\right)$$

where *c_i_* is the atomic fraction of the corresponding element, *R* is the gas constant, 8.314 JK^−1^ mol^−1^ and *n* is the number of component elements [187].

The total configurational entropy in an ideal solid solution is therefore defined as being less than 0.69R for low entropy alloys, between 0.69R and 1.61R for medium entropy alloys, and greater than 1.61R for high entropy alloys. The atoms in the alloys are assumed to be at random positions within the structure in this definition, which is uncommon in metallic solutions. Based on the composition, some non-equiatomic alloys are nonetheless regarded as HEA, even though their configuration entropy is less than 1.61R. Although the entropy-based definition does not require it, a different frequent interpretation is that HEA must be composed of a single-phase solid solution [188,189].

Despite the fact that multicomponent alloys are frequently referred to as “high entropy alloys”, they may not always show high entropy. Although they do exhibit a limited number of phases, high entropy effects are only partially responsible [183]. Additionally, intermetallic compounds are not always detrimental, and the requirement that HEA must be composed of single-phase solid solutions (BCC, FCC, or HCP) may be somewhat restrictive. As a result, new names for multicomponent alloys, such as multi-principal alloys (MPEA) and complex concentrated alloys (CCA), began to be employed [188,190].

The principal characteristics of HEA, besides high entropy, are the sluggish diffusion effect, the lattice distortion effect, and the cocktail effects. All these are described in several papers [188,191,192]. The sluggish diffusion refers to the circumstances in HEAs, where atoms might not achieve vacancies so easily, because of different bonds that are formed between the different atoms, creating a complex energy landscape. The lattice distortion effect is caused by unsymmetrical bonding between atoms with different atomic sizes and electronic structures, affecting the physicochemical properties of the material. The different elements from these alloys are also responsible for the cocktail effect, which means that the combination of elements can create properties that the individual elements do not possess; moreover, in some cases the effect can even be opposed, such as when a soft element is added into a mixture and helps to harden the alloy [193,194].

These new alloys show promising results in various sectors. According to their composition, they can be divided into metallic HEAs and ceramic HEAs. The metallic HEAs include the Cantor-based, which consists of the FeCoNiMnCr series and refractory alloys, composed of elements such as Ti, Mo, Ta, Zr, Hf, V, Nb, etc., having improved mechanical properties, being designed for high-temperature applications and corrosive environments. The ceramic HEAs consist of metal nitrides and carbides of transition elements, possessing high strength, thermal stability, and good corrosion resistance [195,196].

Refractory high entropy alloys (RHEAs) are a new and promising category of alloys. These alloys may replace the conventional ones, such as Ni-based superalloys or SiC, being well suited for use in extreme environments such as nuclear and aerospace applications [197,198,199]. The mechanisms of high-temperature oxidation of HEAs are presented in a recent review by Veselkov et al. [200].

### 3.1. Selection of Alloys Composition

Although nuclear power is a great alternative energy supply, being carbon-free, it still has its shortcomings, such as radioactive waste and high capital costs. However, future generations of nuclear reactors, both Gen IV fission reactors and fusion reactors, should be able to sustainably increase efficiency, minimize nuclear waste and reduce costs [201,202]. The challenges here are to find suitable materials that are capable of withstanding the harsh environment to which these materials would be exposed in this type of reactor. Fortunately, the discovery of HEAs opened a new door in the world of material science and their research is exponentially increasing. There are already auspicious materials, such as WTaCrV and combinations of Ta-Ti-V-Zr-Hf-W or Co-Cr-Ni-Fe-Mn [202].

The conditions at which future advanced systems, such as generation IV systems [203] and fusion nuclear reactors, are expected to operate are much higher than current systems. The six-generation IV systems will be capable of reaching temperatures of at least 500 °C, but go as far as 1000 °C. In addition to this, exposure to radiation and coolants such as liquid metals, liquid salts, or gas creates a very harsh environment in which the exposed material can degrade due to corrosion, stress, vibration, irradiation, etc. [204]. On the other hand, fusion reactors face even harsher conditions, having neutrons with ten times more energy, compared with fission reactors, while some components have to resist plasma temperatures and He embrittlement [202].

Future nuclear reactors using HEAs will confront two major challenges: low economic efficiency and significant radioactivity. Due to the small amount of spent fuel, during the fusion process, induced radioactive materials are the primary source of the reactor’s radioactive waste. The challenges and costs associated with managing these materials after their use will increase due to the radioactive isotopes’ lengthy half-lives. Therefore, another requirement for the components of ideal HEAs is to have minimal induced radioactivity [205,206,207]. However, a lot of HEAs contain unwanted high-activation elements, such as cobalt, which can transform into ^60^Co when exposed to neutrons [208,209].

Despite the fact that methodologies for designing HEAs with tailored properties are still in their infancy, and most of the actual discoveries were based on assumptions and trial and error, Tan et al. have recently applied density functional theory (DFT) to screen for the possible formation of single-phase multicomponent alloys based on low activation elements, synthesizing the TaTiVW alloy [210]. Other low-activation HEAs were synthesized starting from the idea of replacing the high-activation element Nb from the TiVNbTa alloy, forming TiVZrTa and TiVCrTa. These new alloys showed significantly less irradiation-induced damage while maintaining similar indentation hardness and modulus compared to the TiVNbTa alloy [211]. CALPHAD databases may also be useful in assessing possible microstructures, but they should be carefully used as the abundance of phases can be poorly predicted [212]. An alternative method that may become more widely used in the future was described by Huang et al., who employed a machine learning algorithm to obtain relatively high accuracy in predicting phase selection in HEAs [213].

One possible approach to mitigating the high cost associated with HEAs would be to use traditional materials coated with HEAs. Both monolayers of AlCrMoNbZr and (AlCrMoNbZr)N, as well as a multilayer composed of these two with equal thickness, were deposited on Si substrate and subjected to He^+^ irradiation (400 keV, 8 × 10^16^ ion/cm^2^), showing that a thickness of 50 nm provided adequate stability and corrosion resistance [214].

The limitations placed on certain nuclear environments (such as the requirement for low activation or low neutron cross-section) may limit the elemental palettes, but the increased compositional freedom provided by HEAs still presents a special opportunity for the development of alloys for advanced nuclear applications. This particularly potent potential exists in the tweaking of compositions across a broad range to optimize certain irradiation responses [215].

### 3.2. Irradiation Damage

Compared to the traditional alloys that are widely used in Gen II and Gen III reactors as fuel cladding, due to their high resistance to corrosion and low neutron absorption, Gen IV reactors also require withstanding much greater temperatures, at which these alloys may exhibit increased irradiation-induced defects [194,202].

The main effects in these conditions are represented by atomic displacements, irradiation induced hardening, irradiation induced creep, material swelling, and stress corrosion cracking [216]. Therefore, the main strategy for designing materials with high resistance to radiation consisted of introducing high density of point defects. However, it is difficult to maintain a high dislocation density at increased temperatures during high-dose irradiation [208]. The main effects of irradiation damage are produced by neutrons with over 1.0 MeV. These effects are the ionization effects, when electrons are dislocated from their orbits; nuclear transmutation reactions, when nuclei are transformed as a result of neutron impact; displacement damage effects, when atoms are displaced from their initial lattice; and phase transformation effects, when the ordered phase transforms into a disordered phase or amorphous [217].

Due to HEAs compositional complexity and increased atomic stress, amorphization is facilitated. Moreover, having decreased electron-free paths, the thermal spikes caused by particle irradiation are prolonged, favoring recombination. These mechanisms are regarded as self-healing mechanisms [218]. For the quantification of radiation damage, an improved model was recently presented by Nordlund et al. [219].

By promoting recombination and annihilation of defect mobility processes and defect production, both heat dissipation and mechanical properties are being improved. RHEA, in particular, is promising due to its stable mechanical properties even at very high temperatures [204]. The resistance to irradiation can be appropriately evaluated through molecular dynamic simulations helping, thus, to evaluate the defect evolution as well as the size and distribution of defects [207,220]. Regarding the study of the irradiation effects, such as swelling and hardening, the most common method is the ion irradiation method. When irradiated with 300 keV Ni^+^ (1.5 × 10^16^ ions/cm^2^), the HfNbTaTiZr RHEA displayed significantly less swelling and hardening effects than traditional nuclear materials [221]. Also, for the HfTaTiVZr RHEA, it was observed to have around 20% hardening compared to 50% hardening for 304 stainless steel alloys under heavy ion exposure (4.4 MeV, 1.08 × 10^17^ Ni^2+^ ions/cm^2^) [206]. Other RHEA, such as MoNbTaTi, MoNbTaW, and MoNbTaTiW are also currently being investigated, showing increased irradiation resistance [222].

Currently, climate change and the continuous demand for energy have reawakened interest in nuclear energy, as it represents the most efficient and reliable source of clean energy without carbon emissions. At the International Conference on Climate Change and the Role of Nuclear Power held in September 2019, it was established that a new nuclear concept named Small Modular Reactors (SMRs) could be a viable option for nuclear energy contribution to mitigating climate change [223].

Along with generation IV nuclear reactors, SMRs are new generation reactors with a power generation capacity of up to 300 MW per unit (around a third of the capacity of traditional reactors), of small size and, have one or multiple modules. That is precisely why it is considered that the benefits of an SMR derive from their small size, easy assembly, modularity, and the ability to be located in areas where large power plants cannot be built [224].

An ideal SMR concept should be durable, secure, proliferation resistant, simple to build, easy to use, and affordable.

This technology is designed to save on cost and construction time and to be deployed incrementally to match increasing energy demand.

There are three major groups of small modular reactor designs that are actively being developed around the world. One group of SMRs is based on the design concept of PWRs, proven, and widely utilized light water reactors. Another group is based on the design of gas-cooled SMRs, while the last group follows the concept of advanced reactors of type Gen IV which use liquid metal or liquid salt as coolants. This group is expected to be the most difficult to license, because, until now, there is not much experience in operating or providing facilities for testing new designs [224].

However, there are still many challenges that SMR technology needs to overcome before it can achieve commercial deployment. These challenges are to be addressed by research and development, such as improving the performance of structural materials, testing and validating technological improvements in components and systems, testing and fabricating fuel, and addressing regulatory (defining the source term for multi-module SMR plants and the emergency planning zone), and licensing issues. Other issues to be addressed are the spent nuclear fuel, the costs of reactor decommissioning, and the development of new codes and standards. From the early design stage, the development of SMRs regarding the entire life cycle and the associated spent fuel management, radioactive waste management, and decommissioning responsibilities must be considered.

Another product called the Micro Modular Reactor (MMR) is a specific type of SMR technology. MMR is being licensed in Canada and the U.S.A. as the first “fission battery” in commercialization, and the demonstration units are scheduled for the first nuclear power in 2026. Like batteries, multiple MMR units can be linked together to provide as much power as needed. Multiple MMR units can power communities in isolated areas, large industrial sites, and cities. The modules can be combined in different ways for different sites and needs, including integration with renewable microgrids. It is estimated that a MMR can produce approximately 15 MW (thermal) of process heat to generate electrical power and/or heat, and operate for 20 years without refueling.

The MMR will use a new type of fuel namely Fully Ceramic Micro-Encapsulated (FCM™) fuel that replaces the graphite matrix around the typical TRISO coated fuel. This creates an extra barrier to fission product release and improves each TRISO particle’s structural and containment characteristics. For manufacturing this specific fuel, a 3D-printing process was recently licensed by USNC from ORNL [225]. Because the MMR will be fueled only once in its lifetime, there will not be problems with waste management.

A significant MMR advantage is the fully modularized construction approach and that MMRs use proven equipment and components that are commercially available.

The MMR has incomparable tolerance to out-of-normal conditions, whether they are natural hazards, such as flooding and earthquakes, or human actions (operator errors or deliberate sabotage). The catastrophic accidents for traditional or other advanced reactors are minor affairs for the MMR—even when scenarios strike simultaneously [225]. Synergistic studies are needed to realistically evaluate the behavior of the alloys, where they are exposed both to the corrosive medium and to irradiation [226].

## 4. Conclusions

It is difficult to present a firm conclusion for such a complex matter as materials development for nuclear power. In fact, each subchapter of this paper is an approach with some conclusion. It is clear, however, that future nuclear energy systems will require materials that can withstand very aggressive and complex environments with very high operating temperatures, higher levels of irradiation, and possible aggressive coolants. The advance of future fission and fusion nuclear reactors depends on the irradiation response of such materials. It is important for the materials used to have reduced activation and a low neutron absorption cross-section, so they can be safely recycled in a timely manner after decommissioning.

The need to increase alloy performance by modifying the composition and elaborating and testing novel coatings on Zr alloys and stainless steel is a part of a recent trend. For future generation reactor systems, high entropy alloys have been proposed, presenting their composition selection and irradiation damage.

Additionally, in our time of great uncertainties, with complex social, environmental, and political factors having an influence on energy type selection, any challenge in this field is based on the concept of increased security and performance for materials with more investigations into fundamental and applied science.

## Figures and Tables

**Table 1:** Leading institutes and their options on ATF claddings.

Leading Institute	Agreed Research Directions
Oak Ridge National Laboratory	FeCrAl, SiCf/SiC, polycrystalline nanolaminates of ternary carbides and nitrides (MAX phases), coated Zr alloy
Los Alamos National Laboratory	FeCrAl, Mo alloy
Westinghouse Electric Corporation	Coated Zr alloy, SiCf/SiC
General Electric Company	FeCrAl
Electric Power Research Institute	Mo-alloy
Framatome	Coated Zr alloy
French Alternative Energies and Atomic Energy Commission	Coated Zr alloy, SiCf/SiC
Korea Atomic Energy Research Institute	Coated Zr alloy, SiCf/SiC
Japan Atomic Energy Agency	FeCrAl, SiCf/SiC
Nuclear Power Institute of China	Coated Zr alloy, FeCrAl, SiCf/SiC

**Table 2:** Proposed coatings for accident tolerant fuels (ATF) cladding.

Type	Coating	Mechanical Properties	Resistance to Corrosion
Metals	Cr, CrAl, AlTiCr, FeCrAl, 310 SS, high-entropy alloys	High temperature strength Creep resistance	Improved oxidation/corrosion resistance compared to Zr due to multiphase oxide formation
Carbides	MAX phase carbides (Cr_2_AlC, Zr_2_AlC, Ti_2_AlC, Ti_3_SiC_2_), Cr_x_C_y_, SiC	High melting point Low chemical reactivity	Can provide superior oxidation resistance, but unstable oxide growth and the formation of oxide scales were also observed
Nitrides	CrN, AlCrN, TiAlN, TiAlCrN, TiAlSiN	Good coating adhesion Minimal coating spallation after deformation Improve the overall stiffness of the cladding	Overall improved resistance to corrosion, but cracking and formation of second phases or Ti-enriched zones with low oxidation resistance was also observed
Silicides	ZrSi_2_	Low density High melting point Linear thermal expansion coefficient and compositional compatibility with the substrate	Oxidation resistance increased two orders of magnitude compared to Zry-4 at 700 °C, for 20 h, without cracking
Multilayer	Cr-Zr/Cr/CrN, CrN/Cr, Cr/CrAl, Cr/FeCrAl, Mo/FeCrAl, TiN/TiAlN	More resistant to cracking under high temperature ramps than single layers	Can provide increased corrosion resistance due to self-healing ability

## Data Availability

Not applicable.
